# The association between reasons for first using cannabis, later pattern of use, and risk of first-episode psychosis: the EU-GEI case–control study

**DOI:** 10.1017/S0033291723001071

**Published:** 2023-11

**Authors:** Edoardo Spinazzola, Diego Quattrone, Victoria Rodriguez, Giulia Trotta, Luis Alameda, Giada Tripoli, Charlotte Gayer-Anderson, Tom P Freeman, Emma C Johnson, Hannah E Jongsma, Simona Stilo, Caterina La Cascia, Laura Ferraro, Daniele La Barbera, Antonio Lasalvia, Sarah Tosato, Ilaria Tarricone, Giuseppe D'Andrea, Michela Galatolo, Andrea Tortelli, Ilaria Tagliabue, Marco Turco, Maurizio Pompili, Jean-Paul Selten, Lieuwe de Haan, Paulo Rossi Menezes, Cristina M Del Ben, Jose Luis Santos, Manuel Arrojo, Julio Bobes, Julio Sanjuán, Miguel Bernardo, Celso Arango, James B Kirkbride, Peter B Jones, Michael O'Donovan, Bart P Rutten, Jim Van Os, Craig Morgan, Pak C Sham, Isabelle Austin-Zimmerman, Zhikun Li, Evangelos Vassos, EU-GEI WP2 Group, Robin M Murray, Marta Di Forti

**Affiliations:** 1 Department of Psychosis Studies, Institute of Psychiatry, Psychology and Neuroscience, King's College of London, London, UK; 2 Social, Genetic and Developmental Psychiatry Centre, Institute of Psychiatry, Psychology and Neuroscience, King's College London, London SE5 8AF, UK; 3 National Institute for Health Research, Mental Health Biomedical Research Centre at South London and Maudsley NHS Foundation Trust and King's College, London, UK; 4 South London and Maudsley NHS Mental Health Foundation Trust, London, UK; 5 Service of General Psychiatry, Treatment and Early Intervention in Psychosis Program, Lausanne, University Hospital (CHUV), Lausanne, Switzerland; 6 Centro Investigacion Biomedica en Red de Salud Mental (CIBERSAM); Instituto de Biomedicina de Sevilla (IBIS), Hospital Universitario Virgen del Rocio, Departamento de Psiquiatria, Universidad de Sevilla, Sevilla, Spain; 7 Biomedicine, Neuroscience and Advanced Diagnostic Department, Psychiatry Section, University of Palermo, Palermo, Italy; 8 Department of Health Service and Population Research, Institute of Psychiatry, Psychology and Neuroscience, King's College London, London, UK; 9 Addiction and Mental Health Group (AIM), Department of Psychology, University of Bath, Bath, UK; 10 National Addiction Centre, Institute of Psychiatry, King's College London, London, UK; 11 Department of Psychiatry, Washington University School of Medicine, St. Louis, MO, USA; 12 Psylife Group, Division of Psychiatry, University College London, London, UK; 13 Department of Mental Health and Addiction Services, ASP Crotone, Crotone, Italy; 14 Section of Psychiatry, Department of Neuroscience, Biomedicine and Movement Sciences, University of Verona, Verona, Italy; 15 Department of Medical and Surgical Science, Psychiatry Unit, Alma Mater Studiorum Università di Bologna, Bologna, Italy; 16 Institut Mondor de recherché biomedicale, Creteil, France; 17 Etablissement Public de Sante Maison Blanche, Paris, France; 18 Department of Medicine and Surgery, University of Milano Bicocca, Monza, Italy; 19 Department of Mental Health and Addiction Services, ASST Lecco, Lecco, Italy; 20 Department of Neurosciences, Mental Health and Sensory Organs, Suicide Prevention Center, Sant'Andrea Hospital, Sapienza University of Rome, Rome, Italy; 21 Rivierduinen Institute for Mental Health Care, Leiden, The Netherlands; 22 Department of Psychiatry and Neuropsychology, School for Mental Health and Neuroscience, South Limburg Mental Health Research and Teaching Network, Maastricht University Medical Centre, Maastricht, The Netherlands; 23 Early Psychosis Section, AmsterdamUMC, Academic Medical Centre, University of Amsterdam, Meibergdreef 5, 1105 AZ Amsterdam, The Netherlands; 24 Department of Preventive Medicine, Faculdade de Medicina, Universidade of São Paulo, São Paulo, Brazil; 25 Department of Psychiatry, Servicio de Psiquiatría Hospital “Virgen de la Luz”, Cuenca, Spain; 26 Department of Psychiatry, Psychiatric Genetic Group, Instituto de Investigación Sanitaria de Santiago de Compostela, Complejo Hospitalario Universitario de Santiago de Compostela, Santiago, Spain; 27 Department of Medicine, Psychiatry Area, School of Medicine, Universidad de Oviedo, Centro de Investigación Biomédica en Red de Salud Mental (CIBERSAM), Oviedo, Spain; 28 Department of Psychiatry, School of Medicine, Universidad de Valencia, Centro de Investigación Biomédica en Red de Salud Mental (CIBERSAM), Valencia, Spain; 29 Barcelona Clinic Schizophrenia Unit, Neuroscience Institute, Hospital Clinic of Barcelona, University of Barcelona, Institut d'Investigacions Biomèdiques August Pi I Sunyer (IDIBAPS), Biomedical Research Networking Centre in Mental Health (CIBERSAM), Barcelona, Spain; 30 Department of Child and Adolescent Psychiatry, Institute of Psychiatry and Mental Health, Hospital General Universitario Gregorio Marañón, School of Medicine, Universidad Complutense, IiSGM, CIBERSAM, Madrid, Spain; 31 Reader; Psylife Group, Division of Psychiatry, University College London, London, UK; 32 Department of Psychiatry, University of Cambridge, Cambridge, UK; 33 CAMEO Early Intervention Service, Cambridgeshire & Peterborough NHS Foundation Trust, Cambridge, UK; 34 Division of Psychological Medicine and Clinical Neurosciences, MRC Centre for Neuropsychiatric Genetics and Genomics, Cardiff University, Cardiff, UK; 35 Department Psychiatry, Brain Centre Rudolf Magnus, Utrecht University Medical Centre, Utrecht, The Netherlands; 36 Department of Psychiatry, Centre for PanorOmic Sciences, and State Key Laboratory of Brain and Cognitive Sciences, Li KaShing Faculty of Medicine, The University of Hong Kong, Hong Kong, China; 37 Research Foundation, National Institute for Health Research Biomedical Research Centre at South London and Maudsley NHS Foundation Trust and King's College London and the NIHR BRC at University College London, London, UK

**Keywords:** Cannabis use, path analysis, psychotic disorders

## Abstract

**Background:**

While cannabis use is a well-established risk factor for psychosis, little is known about any association between reasons for first using cannabis (RFUC) and later patterns of use and risk of psychosis.

**Methods:**

We used data from 11 sites of the multicentre European Gene-Environment Interaction (EU-GEI) case–control study. 558 first-episode psychosis patients (FEPp) and 567 population controls who had used cannabis and reported their RFUC.

We ran logistic regressions to examine whether RFUC were associated with first-episode psychosis (FEP) case–control status. Path analysis then examined the relationship between RFUC, subsequent patterns of cannabis use, and case–control status.

**Results:**

Controls (86.1%) and FEPp (75.63%) were most likely to report ‘because of friends’ as their most common RFUC. However, 20.1% of FEPp compared to 5.8% of controls reported: ‘to feel better’ as their RFUC (χ^2^ = 50.97; *p* < 0.001). RFUC ‘to feel better’ was associated with being a FEPp (OR 1.74; 95% CI 1.03–2.95) while RFUC ‘with friends’ was associated with being a control (OR 0.56; 95% CI 0.37–0.83). The path model indicated an association between RFUC ‘to feel better’ with heavy cannabis use and with FEPp-control status.

**Conclusions:**

Both FEPp and controls usually started using cannabis with their friends, but more patients than controls had begun to use ‘to feel better’. People who reported their reason for first using cannabis to ‘feel better’ were more likely to progress to heavy use and develop a psychotic disorder than those reporting ‘because of friends’.

## Introduction

A meta-analysis including cross-sectional and longitudinal studies has shown that heavy cannabis use is associated with a 4-fold increase in the risk of psychosis (Marconi, Di Forti, Lewis, Murray, & Vassos, 2016). Moreover, a higher incidence of psychotic disorders was reported in regions with a higher prevalence of daily use and greater availability of high-potency cannabis (Di Forti et al., 2019). The average proportion of delta-9-tetrahydrocannabinol (THC) in the cannabis available in international markets increased from 1970 to 2017 (Freeman et al., 2021). Two studies conducted in Denmark found that an increase in the use and potency of cannabis in the population during the past two decades was accompanied by a noticeable increase in the incidence of cannabis-induced psychosis (Hjorthoj, Larsen, Starzer, & Nordentoft, 2021*a*), and a 3- to 4-fold increase in the proportion of cases of schizophrenia associated with cannabis use disorder (CUD) (Hjorthoj, Posselt, & Nordentoft, 2021*b*). Moreover, a recent within-person analysis – where each participant serves as their control – found that cannabis is likely to have a causal impact on psychosis, but not the other way around (van Os et al., 2021).

Some argue that the association between cannabis use and psychotic disorders can be explained as an attempt to ‘self-medicate’ by those who experience sub-clinical psychosis, trying to relieve existing subthreshold psychotic symptoms (Khantzian, 1985). However, most studies report that patients with psychosis use cannabis for the same reasons as the general population, such as for the pleasurable effects and social reasons rather than to self-medicate (Dekker, Linszen, & De Haan, 2009; Dekker et al., 2010; Green, Kavanagh, & Young, 2004; Kolliakou, Joseph, Ismail, Atakan, & Murray, 2011; Pérez, Santacana, Baquero, & Pérez-Solà, 2014).

While some studies have examined why people use cannabis and whether the subjective effects of cannabis differ between patients with their first episode of psychosis and controls (Bianconi et al., 2016; Dekker, Koeter, Van Den Brink, & Investigators, 2012; Dekker et al., 2009; Peters et al., 2009), an accurate search of the literature (see online supplementary) indicated little evidence on the reasons for the first use of cannabis (RFUC). Moreover, it is not clear if RFUC explains later patterns of use, or if RFUC differs between those who later develop psychosis and healthy controls.

This is the first study that uses data from a large multisite first-episode psychosis (FEP) case–control design and aims to examine (1) the socio-demographic factors associated with RFUC and (2) which RFUC, among cannabis users, is associated with harmful patterns of cannabis use and with an increased risk for psychotic disorders.

## Methods

### Sample

This investigation is based on the European network of national schizophrenia networks studying Gene-Environment Interactions (EU-GEI study, http://www.eu-gei.eu), a multicentre incidence and case-sibling-control study of genetic and environmental determinants of psychotic disorder (Gayer-Anderson et al., 2020; Jongsma et al., 2018) consisting of first-episode psychosis patients (FEPp) and population-based controls recruited between 2010 and 2015, who provided information on cannabis use (Gayer-Anderson et al., 2020). Both FEPp and controls were collected in 17 catchment areas in six countries: Southeast London, Cambridgeshire and Peterborough (England); central Amsterdam, Gouda and Voorhout (the Netherlands); part of the Veneto region, Bologna municipality, city of Palermo (Italy); 20th Arrondissement of Paris, Val-de-Marne, Puy-de-Dôme (France); Madrid (Vallecas), Barcelona, Valencia, Oviedo, Santiago, Cuenca (Spain); and Ribeirão Preto (Brazil).

### Participants

All patients presenting with FEP who were referred to the mental healthcare services within the 17 catchment areas were identified by trained researchers. Patients were included in the current study if they were aged 18–64 years and resident within the study areas at the time of their first presentation and if they presented with a clinical diagnosis for an untreated FEP (*International Statistical Classification of Diseases, Tenth Revision* (*ICD-10*) codes F20–F33). Exclusion criteria were: (a) previous contact with psychiatric services for psychosis; (b) evidence of psychotic symptoms precipitated by an organic cause; and (c) transient psychotic symptoms resulting from acute intoxication, as defined by the ICD-10 (codes F1X.5). Controls were recruited using quota sampling based on local census data to ensure samples representativeness in each catchment area's population at risk in terms of age, gender, and ethnicity. Inclusion criteria for controls were: (a) aged between 18 and 64 years; (b) resident within the catchment areas at the time of consent into the study; (c) sufficient command of the primary language at each site to complete the assessments; and (d) no current or past psychotic disorder. The authors assert that all procedures contributing to this work comply with the ethical standards of the relevant national and institutional committees on human experimentation and with the Helsinki Declaration of 1975, as revised in 2008. All participants who agreed to take part in the study provided informed, written consent; and ethical approval was provided by research ethics committees in each site: South London and Maudsley and Institute of Psychiatry Research Ethics Committee; National Research Ethics Service Committee East of England–East Cambridge; Medisch-Ethische Toetsingscommissie van het Academisch Centrum te Amsterdam; Comité Ético de Investigación Clínica Hospital Gregorio Marañón; Comité Ético de Investigación Clínica del Hospital Clinic de Barcelona; Comité Ético de Investigación Clínica del Hospital Clinic Universitari de Valencia; Comité Ética de la Investigación Clínica del Principado de Asturias; Comité Ético de Investigación Clínica de Galicia; Comité Ético de Investigación Clínica del Hospital Virgen de la Luz de Cuenca; Comité de Protéction des Personnes–CPP Île de France IX; Comitato Etico Policlinico S Orsola Malpighi; Comitato Etico Azienda Ospedaleria Universitaria di Verona; Comitato Etico Palermo 1, Azienda Ospedaliera Policlinico ‘Paolo Giaccone’; and Research Ethics Committee of the clinical Hospital of Ribeirão Preto Medical School, University of São Paulo, Brazil.Further information on the general study methods is available in the EU-GEI core paper on the description of the objectives and main aspects of the study (Gayer-Anderson et al., 2020).

### Measures and assessments

Data on age, gender, and self-reported ethnicity were collected using a modified version of the Medical Research Council Sociodemographic Schedule (Mallett, Leff, Bhugra, Pang, & Zhao, 2002). Diagnoses of psychotic disorders were confirmed using the OPerational CRITeria (OPCRIT) system (McGuffin, Farmer, & Harvey, 1991; Williams, Farmer, Ackenheil, Kaufmann, & McGuffin, 1996), which was completed by centrally trained investigators whose inter-rater reliability was assessed throughout the study (*κ* = 0.7). Family history of mental illness and parental history of psychosis were collected using the Family Interview for Genetic Studies questionnaire (Maxwell, 1992). Finally, the Intelligence Quotient (IQ) score was derived using the short form of the WAIS-III (IQ), including selected items of the following subtests: digit symbol coding (a measure of processing speed), arithmetic (working memory), block design (visuospatial processing), and information (verbal knowledge) (Tripoli et al., 2021).

Detailed information on cannabis use was collected using the latest version of the Cannabis Experiences Questionnaire (Barkus, Stirling, Hopkins, & Lewis, 2006), for the EU-GEI study (CEQ_EU−GEI_). Consistently with Di Forti et al. (2019), we used the combined measure of frequency and type of cannabis used derived from the following variables: (1) Frequency of use: never or occasional = 0, more than once a week = 1, daily = 2; (2) low potency cannabis with less than 10% of THC = 0, high potency THC 10% or greater = 1 (see online supplementary materials for a detailed description of this variable). This combined measure was used to define the *harmful pattern of use* and coded as follows: occasional use with any potency of use = 1, more than once a week and low potency = 2, more than once a week and high potency = 3, daily or almost and low potency = 4, daily or almost and high potency = 5 (see online supplementary). Age at first cannabis use was considered as a continuous numerical variable.

The information on RFUC was derived from the responses to the question ‘why did you first try cannabis?’ in the form of the following multiple-choice: (a) my friends were using it; (b) my family members were using it; (c) to feel better (to get relief from either physical or psychological discomfort) and (d) other. Subjects were able to provide up to 4 RFUC.

### Statistical analysis

We compared the number of different reported RFUC in cases and controls, also taking into account the overlapping answers.

T-test (Mann–Whitney where appropriate) and χ^2^ in STATA16 were used to test for association between socio-demographics and IQ with RFUC. Only the variables associated with RFUC were included as covariates in the Path analyses.

We conducted unadjusted and adjusted logistic regressions using STATA-16 to examine whether RFUC was associated with FEP case–control status, adjusting for IQ score, gender, age at first cannabis use, ethnic minority status, and the harmful pattern of cannabis use. We conducted the analysis in stages to test four models. Model A examined the unadjusted associations between different RFUC and FEP case–control status. Model B examined the associations after adjusting for the harmful patterns of use. Model C additionally adjusted for age at first cannabis use. Lastly, model D additionally adjusted for ethnic minority status, IQ score, and gender. We compared the statistical fit of the four models using Log-Likelihood (LL), Akaike information criteria (AIC), and Bayesian information criteria (BIC).

Path analysis (Stage, Carter, & Nora, 2004) was run in STATA-16 to test the hypothesised direction in the relationship between multiple RFUC as predictors, with (1) pattern of cannabis use and (2) FEP case–control status.

In the regression analyses and in the path model, we included the participants who reported to have used cannabis either because of ‘friends’, ‘family’, or ‘to feel better’.

## Results

### Socio-demographic characteristics

Participants were recruited and consented to the study between May 1, 2010, and April 1, 2015. The 20th arrondissement of Paris was the only one that did not contribute to the recruitment of population controls; hence, it was excluded from the analyses. As in a previous EU-GEI paper (Di Forti et al., 2019), we also excluded Verona, Santiago, Oviedo, Valencia, and Cuenca because they had at least 10% of data missing on the measures of cannabis use. This left 901 FEPp and 1235 controls (see recruitment flow chart in the online supplementary materials).

585 FEPp (64.93%) and 574 (46.4%) of the controls reported lifetime cannabis use. Among these, 27 FEPp (4.62%) and 7 controls (1.22%) were excluded because of missing data on RFUC. Therefore, the sample resulted in a total size of 558 FEPp and 567 controls.

**Figure 1.**
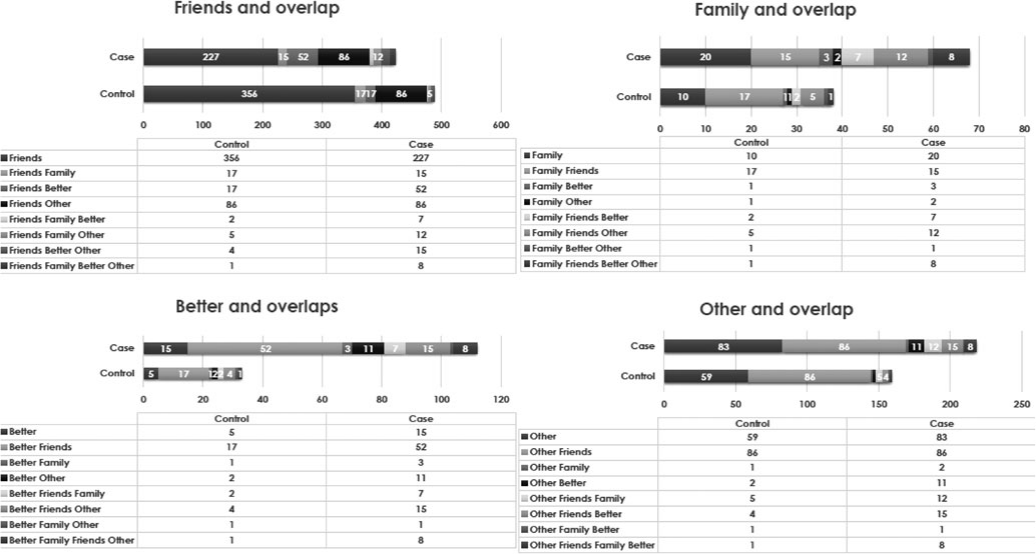
Overlapping reported RFUC in cases and controls.

We found that, among users, 488 (86.1%) controls and 422 (75.63%) FEPp reported RFUC because of ‘friends’, while 38 (6.70%) controls and 69 (12.37%) FEPp reported RFUC because of their ‘family’ members; 33 (5.82%) controls and 112 (20.07%) of FEPp started ‘to feel better’, and 159 (28.04%) controls and 218 (39.07%) of FEPp because of ‘other’ reasons. See Fig. 1 for detailed information on the overlapping answers for this variable.

Cannabis users who reported RFUC because of ‘friends’ did not show any association with the socio-demographic variables assessed. However, those who reported RFUC ‘to feel better’ were more often from ethnic minorities (χ^2^(1) = 13.67; *p* < 0.001) and had lower IQ scores (*U* = 6.66; *p* < 0.001). Similarly, those who reported RFUC because of their ‘family’ members, were more often from ethnic minorities (χ^2^(1) = 7.97; *p* = 0.005) and had lower IQ scores (*U* = 3.11; *p* = 0.002). Lastly, those who reported RFUC ‘other’ reasons were more likely to be male (χ^2^(1) = 8.65; *p* = 0.003) and were more often from ethnic minorities (χ^2^(1) = 4.43; *p* = 0.035; Table 1).

**Table 1.** Descriptive sociodemographic analyses based on reported RFUC (in cannabis users)

		‘Friends’ yes/no N (% or SD)	Statistics and *p* value	‘Family’ yes/no N (% or SD)	Statistics and *p* value	‘To feel better’ yes/no N (% or SD)	Statistics and *p* value	‘Other’ yes/no n (% or SD)	Statistics and *p* value
Gender	Male	560 (61.54)	χ^2^ = 0.84 *p* = 0.36	71 (66.36)	χ^2^ = 0.86 *p* = 0.35	97 (66.90)	χ^2^ = 1.58 *p* = 0.21	257 (68.17)	χ^2^ = 8.65 *p* = 0.003
Ethnic minority status	Yes	254 (27.94)	χ^2^ = 0.49 *p* = 0.48	43 (40.19)	χ^2^ = 7.97 *p* = 0.005	60 (41.38)	χ^2^ = 13.67 *p* < 0.001	122 (32.45)	χ^2^ = 4.43 *p* = 0.035
Ethnicity	White	655 (72.06) 115 (12.65) 71 (7.81) 22 (2.42) 46 (5.06)	χ^2^ = 2.96 *p* = 0.57	64 (59.81) 21 (19.63) 16 (14.95) 1 (0.93) 5 (4.67)	χ^2^ = 15.88 *p* = 0.003	85 (58.62) 26 (17.93) 21 (14.48) 4 (2.76) 9 (6.21)	χ^2^ = 17.38 *p* = 0.002	254 (67.55) 48 (12.77) 42 (11.17) 13 (3.46) 19 (5.05)	χ^2^ = 10.41 *p* = 0.034
	Black								
	Mixed								
	Asian								
	Other								
IQ		97.30 (20.25)	*U* = −1.95 *p* = 0.051	91.08 (19.89)	*U* = 3.11 *p* = 0.002	86.10 (17.25)	*U* = 6.66 *p* < 0.001	96.35 (19.15)	*U* = 0.59 *p* = 0.56
Parental mental illness	Yes	310 (34.33)	χ^2^ = 0.02 *p* = 0.88	42 (39.25)	χ^2^ = 1.28 0.26	51 (35.66)	χ^2^ = 0.12 *p* = 0.73	132 (35.58)	χ^2^ = 0.36 *p* = 0.55
Parental Psychosis	Yes	64 (7.09)	χ^2^ = 0.68 *p* = 0.41	11 (10.28)	χ^2^ = 1.54 *p* = 0.21	14 (9.79)	χ^2^ = 1.38 *p* = 0.24	31 (8.36)	χ^2^ = 0.75 *p* = 0.39

χ^2^, Chi-squared test; U, Mann–Whitney U test; *p-value ⩽ 0.05.

FEPp were more likely to belong to ethnic minority groups (χ^2^(1) = 62.37; *p* < 0.001) and had lower IQ (*U* = 14.97; *p* < 0.001) compared to controls (see online supplementary materials).

### Logistic regressions

In the regression and in the path models, we included the participants who reported that they had used cannabis either because of ‘friends’, ‘family’, or ‘to feel better’, thus excluding 59 controls (10.41%) and 83 cases (14.87%) who reported ‘other’ as their RFUC. This resulted in a final sample of 475 FEPp and 508 controls with a complete dataset on reasons to start using cannabis.

The Model A unadjusted logistic regression indicated that RFUC because of ‘friends’ was negatively associated with being a FEP (OR 0.58; 95% CI 0.43–0.79), while those reporting RFUC because of ‘family’ members (OR 1.59; 95% CI 1.03–2.44) or ‘to feel better’ (OR 3.86; 95% CI 2.56–5.82) were more likely to become FEPp. In Model B, after controlling for the harmful patterns of use, RFUC because of ‘family’ members was no longer associated with FEP case–control status, while RFUC because of ‘friends’ was still associated with being a control (OR 0.64; 95% CI 0.45–0.91) and RFUC ‘to feel better’ was still associated with being a FEPp (OR 2.60; 95% CI 1.67–4.04). In Model C, after additionally adjusting for age at first cannabis use, the results remained consistent with Model B for both RFUC because of ‘friends’ (OR 0.62; 95% CI 0.44–0.88) and ‘to feel better’ (OR 2.63; 95% CI 1.69–4.10). In Model D, additionally adjusting for ethnic minority status, IQ score, and gender, RFUC because of ‘friends’ (OR 0.56; 95% CI 0.37–0.83) and ‘to feel better’ (OR 1.74; 95% CI 1.03–2.95) were still associated with FEP case–control status (See Table 2).

**Table 2.** Unadjusted and adjusted associations between RFUC and case–control status

	Case–control outcome			
	Model A R (95% CI)	Model B Adjustment for harmful pattern of use OR (95% CI)	Model C Plus adjustment for age at first cannabis use	Model D Plus adjustment for ethnic minority status and IQ score
Friends	0.58 (0.43–0.79)	0.64 (0.45–0.91)	0.62 (0.44–0.88)	0.56 (0.37–0.83)
Family	1.59 (1.03–2.44)	1.12 (0.69–1.82)	1.12 (0.69–1.81)	0.99 (0.57–1.73)
Better	3.86 (2.56–5.82)	2.60 (1.67–4.04)	2.63 (1.69–4.10)	1.74 (1.03–2.95)

Model A: unadjusted associations; Model B: adjusted for harmful pattern of use; Model C: adjusted for harmful pattern of use and age at first cannabis use; Model D: adjusted for harmful pattern of use, age at first cannabis use, ethnic minority status, and IQ score.

### Path analysis

**Figure 2.**
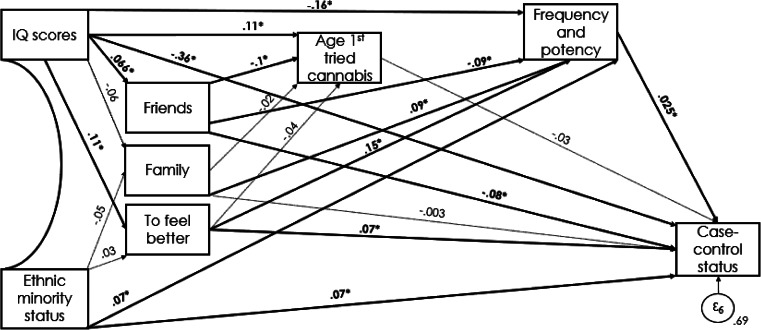
Direct and indirect pathways between IQ, ethnicity, RFUC and case–control status. Significant pathways signified by solid arrows and *; nonsignificant pathways represented by dotted lines. Model fit: χ^2^ = 12.50, RMSEA = 0.040, comparative fit index CFI = 0.99..

The path model had a good fit to the data: root mean square error of approximation (RMSEA) = 0.040, comparative fit index (CFI) = 0.99 (Fig. 2). RFUC because of ‘friends’ was associated with being a control (*β* = −0.08; *p* = 0.002), while ‘to feel better’ was associated with being a FEPp (*β* = 0.07; *p* = 0.014) (see Table 3 for direct associations). Lower IQ scores were indirectly associated with RFUC ‘to feel better’ (*β* = −0.19; *p*⩽0.001), with a younger age at first cannabis use (*β* = 0.11; *p* = 0.001), harmful pattern of cannabis use (*β* = −0.16; *p*⩽0.001) and were directly associated with being a FEPp (*β* = −0.36; *p*⩽0.001). On the contrary, higher IQ scores were associated with RFUC because of ‘friends’ (*β* = 0.07; *p* = 0.043), which was negatively associated with age at first cannabis use (*β* = −0.10; *p* = 0.002).

**Table 3.** Panel A: Standardised Probit Coefficients (β) for the indirect pathways to psychosis with RFUC and pattern of cannabis use as mediators; Panel B Standardised Probit Coefficients (β) for the direct pathways to psychosis

PANEL A. Indirect pathways to psychosis with RFUC and pattern of cannabis use as mediators															
Predictor variables	Via Friends			Via Family			Via to feel better			Via Age at first cannabis use			Via Frequency-potency of cannabis use		
	*β*	s.e.	p	*β*	s.e.	p	*β*	s.e.	p	*β*	s.e.	p	*β*	s.e.	p
IQ scores	0.07	0.03	0.043	−0.06	0.04	0.066	−0.19	0.03	<0.001	0.11	0.03	0.001	−0.16	0.03	<0.001
Ethnic minority Status	N/A	N/A	N/A	−0.05	0.03	0.12	0.03	0.03	0.336	N/A	N/A	N/A	0.07	0.03	0.025
Friends	N/A	N/A	N/A	N/A	N/A	N/A	N/A	N/A	N/A	−0.10	0.03	0.002	−0.09	0.03	0.002
Family	N/A	N/A	N/A	N/A	N/A	N/A	N/A	N/A	N/A	−0.02	0.03	0.49	0.09	0.03	0.003
To feel better	N/A	N/A	N/A	N/A	N/A	N/A	N/A	N/A	N/A	−0.04	0.03	0.209	0.15	0.03	<0.001
PANEL B. Direct pathways to psychosis															

Predictor variables				Case–control status											
				*β*				s.e.				p			
IQ scores				−0.36				0.03				<0.001			
Ethnic minority status				0.07				0.03				0.017			
Friends				−0.08				0.03				0.002			
Family				−0.003				0.03				0.913			
To feel better				0.07				0.03				0.014			
Age at first cannabis use				−0.03				0.03				0.324			
Frequency-potency of cannabis use				0.25				0.29				<0.001			

Note: *β*, Probit coefficient; SE, Standard error; *p*, probability; N/A, Not applicable.

Both RFUC because of ‘family’ (*β* = 0.09; *p* = 0.003) and ‘to feel better’ (*β* = 0.15; *p*⩽0.001) were associated with a more harmful pattern of cannabis use, the latter also with being a FEPp. See Table 3 for direct and indirect associations.

## Discussion

### Principal findings

To the best of our knowledge (see online supplementary materials for details of literature search), this is the first study to examine what reasons underlie first using cannabis and if these reasons are associated with later pattern of cannabis use and the risk to develop a psychotic disorder.

First, our findings indicate that having friends who use cannabis is the most common reason to start using cannabis among both those with FEPp and the controls; although, a higher proportion of FEPp than controls reported ‘to feel better’ as their RFUC (Fig. 1). Second, we provide the first evidence that, (a) compared to those who RFUC with ‘friends’, those who reported starting ‘to feel better’ as their RFUC are much more likely to progress to daily use of high potency cannabis; and (b) those who started to use cannabis because of their ‘friends’ are more likely to have started their use earlier in their life (Table 3).

### Limitations and strengths

Our findings need to be appraised in the context of some limitations. Firstly, the data on cannabis use were collected retrospectively based on self-report. Therefore, they may be open to recall bias. However, studies comparing laboratory data and self-reported information have shown that cannabis users reliably report their frequency of use as well as the type of cannabis they use (Buchan, Dennis, Tims, & Diamond, 2002; Freeman et al., 2014). While it is possible that FEPp are more likely, compared to controls, to retrospectively report ‘to feel better’ as a RFUC to justify their daily use leading to recall bias, the category ‘to feel better’ includes relief from both physical and psychological discomfort, therefore it is not intended to capture self-medication from prodromal symptoms. Moreover, a previous study has shown that cannabis use is associated with increased risk of psychosis even after adjustment for baseline prodromal symptoms (Mustonen et al., 2018). A recent study pointed out that some people might use cannabis to ameliorate anxiety and depressive symptoms (Radhakrishnan et al., 2022). However, the evidence suggests that cannabis use may increase the risk of developing depression and suicidality (Gobbi et al., 2019). A recent large online survey including responses from 27 169 participants from Canada and the USA, showed that 53% of people using cannabis to relieve physical discomfort reported using it to alleviate pain, followed by difficulties with sleep and headache/migraine as reasons to use (Leung et al., 2022). These are physical complaints persisting in nature and it is plausible that the attempt to alleviate them might lead to regular rather than occasional use.

Thirdly, although pathway analysis tests causal inferences and linkages among variables in the context of an a-priori conceptual model, causation cannot be implied. Despite the validity of path analysis in cross-sectional studies has been confirmed (Etain et al., 2017; Kwok, Cheung, Jak, Ryu, & Wu, 2018; Martin, 2011), prospective longitudinal studies will be required to confirm the detected associations, which nevertheless, in our study clearly places first use of cannabis on average at least a decade before the psychosis onset. Furthermore, this is a FEPp-control study which is less likely to lead to recall bias because the participants are recruited near the onset of their psychosis illness.

Finally, the data presented here were collected from a control sample representative of each site's local population at risk (Gayer-Anderson et al., 2020) and from a subset of FEPp representative of the larger incidence sample recruited from each site Mental Health services over the study period (Jongsma et al., 2018). Thus, our findings have the important strength of describing the RFUC reported by individuals with and without psychosis, from different ethnicities across different geographical areas and are therefore generalisable. Moreover, the use of matched controls might have masked the association between sociodemographic variables with RFUC and pattern of cannabis use.

### Comparison with previous research

Previous research indicated that earlier age at first use is more likely to progress to longstanding use, resulting in overall greater exposure to cannabis (Radhakrishnan, Wilkinson, & D'Souza, 2014). Furthermore, data have suggested that individuals who start using cannabis early in adolescence may be at most risk to develop psychotic disorder (Arseneault et al., 2002; Dragt et al., 2010; Korver et al., 2010); one study suggested they might ‘self-medicate’ with cannabis to alleviate initial symptoms (Ferdinand et al., 2005). In contrast, our findings do not show an association between RFUC to ‘feel better’ and starting to use cannabis early in adolescence, which might be explained by our category ‘to feel better’ referring to relief not only of psychological but also physical discomfort. A recent study, in fact, shows how cannabis use for medical reasons is more common among young adults and older age groups rather than adolescence (Leung et al., 2022). Instead, we found that those who reported a younger age at first cannabis use were more likely to have started using cannabis ‘with friends’ compared to the other groups. This is in line with previous evidence looking at reasons to use cannabis which reports social context as the main reason for use (Kolliakou et al., 2011). The high number of cannabis users who start ‘with friends’ is in accord with previous findings indicating that cannabis users with psychosis have better premorbid social functioning compared to patients with psychosis not using cannabis (Ferraro et al., 2021; Ferraro et al., 2020; Ferraro et al., 2013). A possible explanation for this is that cannabis-using patients with psychosis are more socially skilled, and therefore able to obtain the substance than those who are neurodevelopmentally impaired (Murray et al., 2017). We found no evidence that those who report RFUC ‘to feel better’ are more likely to start using cannabis close to their age of psychosis onset.

RFUC because of ‘family members’ is associated with a more harmful pattern of use. This suggests that targeting the family environment could play an important role in delivering interventions for substance misuse and also to disseminate education messages about the risk associated with cannabis use and its harmful effects on mental health.

The existing evidence clearly indicates that daily use especially of high potency cannabis is robustly associated with an increased probability to develop a psychotic disorder (Di Forti et al., 2009, 2015, 2019). While we cannot separate those seeking relief from physical or psychological discomfort, our data suggest that starting to use cannabis ‘to feel better’ is more likely to progress to daily use of high potency cannabis and to later suffer a first episode psychosis.

In conclusion, while only a minority of cannabis users develops psychosis, understanding what drives people to first use cannabis can provide valuable data on (a) how to identify those more likely to develop a harmful pattern of use and design interventions for harm reduction or use cessation tailored to the individual's needs, (b) provide support and close monitoring to those using cannabis for medical reasons to minimise the risks associated with regular use, and (c) design better public health campaigns that are able to reach the individual in the social and family context where first use is most likely to begin.

## Supporting information

Spinazzola et al. supplementary material Spinazzola et al. supplementary material

## Data Availability

The data that support the findings of the study is available on request from the corresponding author, E.S.

## Supplementary material

The supplementary material for this article can be found at https://doi.org/10.1017/S0033291723001071.

## EU-GEI Collaborators

Kathryn Hubbard^1^, Stephanie Beards^1^, Simona A. Stilo^2^, Mara Parellada^3^, David Fraguas^3^, Marta Rapado Castro^3^, Álvaro Andreu-Bernabeu^3^, Gonzalo López^3^, Mario Matteis^3^, Emiliano González^3^, Manuel Durán-Cutilla^3^, Covadonga M. Díaz-Caneja^3^, Pedro Cuadrado^4^, José Juan Rodríguez Solano^5^, Angel Carracedo^6^, Javier Costas^6^, Emilio Sánchez^7^, Silvia Amoretti^8^, Esther Lorente-Rovira^9^, Paz Garcia-Portilla^10^, Estela Jiménez-López^11^, Eva Velthorst^12^, Nathalie Franke^12^, Daniella van Dam^12^, Fabian Termorshuizen^13,14^, Nathalie Franke^13^, Elsje van der Ven^13,14^, Elles Messchaart^14^, Marion Leboyer^15,16,17,18^, Franck Schürhoff^15,16,17,18^, Stéphane Jamain^16,17,18^, Grégoire Baudin^15,16^, Aziz Ferchiou^15,16^, Baptiste Pignon^15,16,18^, Jean-Romain Richard^16,18^, Thomas Charpeaud^18,19,20^, Anne-Marie Tronche^18,19,20^, Pierre-Michel Llorca^20^, Flora Frijda^21,22^, Giovanna Marrazzo^22^, Lucia Sideli^22^, Crocettarachele Sartorio^22,23^, Fabio Seminerio^22^, Camila Marcelino Loureiro^24,25^, Rosana Shuhama^24,25^, Mirella Ruggeri^26^, Chiara Bonetto^26^, Doriana Cristofalo^26^, Domenico Berardi^27^, Marco Seri^27^, Alexander Richards^28^, and Aoife Coghlan^29^.

^1^Department of Health Service and Population Research, Institute of Psychiatry, Kings College London, De Crespigny Park, Denmark Hill, London SE5 8AF, UK.

^2^Department of Psychosis Studies, Institute of Psychiatry, King's College London, De Crespigny Park, Denmark Hill, London SE5 8AF, UK.

^3^Department of Child and Adolescent Psychiatry, Hospital General Universitario Gregorio Marañón, School of Medicine, Universidad Complutense, IiSGM (CIBERSAM), C/Doctor Esquerdo 46, 28007 Madrid, Spain. ^4^Villa de Vallecas Mental Health Department, Villa de Vallecas Mental Health Centre, Hospital Universitario Infanta Leonor / Hospital Virgen de la Torre, C/San Claudio 154, 28038 Madrid, Spain.

^5^Puente de Vallecas Mental Health Department, Hospital Universitario Infanta Leonor / Hospital Virgen de la Torre, Centro de Salud Mental Puente de Vallecas, C/Peña Gorbea 4, 28018 Madrid, Spain.

^6^Fundación Pública Galega de Medicina Xenómica, Hospital Clínico Universitario, Choupana s/n, 15782 Santiago de Compostela, Spain.

^7^Department of Psychiatry, Hospital General Universitario Gregorio Marañón, School of Medicine, Universidad Complutense, IiSGM (CIBERSAM), C/Doctor Esquerdo 46, 28007 Madrid, Spain.

^8^Department of Psychiatry, Hospital Clinic, IDIBAPS, Centro de Investigación Biomédica en Red de Salud Mental (CIBERSAM), Universidad de Barcelona, C/Villarroel 170, escalera 9, planta 6, 08036 Barcelona, Spain.

^9^Department of Psychiatry, School of Medicine, Universidad de Valencia, Centro de Investigación Biomédica en Red de Salud Mental (CIBERSAM), C/Avda. Blasco Ibáñez 15, 46010 Valencia, Spain.

^10^Department of Medicine, Psychiatry Area, School of Medicine, Universidad de Oviedo, Centro de

Investigación Biomédica en Red de Salud Mental (CIBERSAM), C/Julián Clavería s/n, 33006 Oviedo, Spain. ^11^Department of Psychiatry, Servicio de Psiquiatría Hospital “Virgen de la Luz”, C/Hermandad de Donantes de Sangre, 16002 Cuenca, Spain.

^12^Department of Psychiatry, Early Psychosis Section, Academic Medical Centre, University of Amsterdam,

Meibergdreef 5, 1105 AZ Amsterdam, The Netherlands.

^13^Rivierduinen Centre for Mental Health, Leiden, Sandifortdreef 19, 2333 ZZ Leiden, The Netherlands. ^14^Department of Psychiatry and Neuropsychology, School for Mental Health and Neuroscience, South Limburg Mental Health Research and Teaching Network, Maastricht University Medical Centre, P.O. Box 616, 6200 MD Maastricht, The Netherlands.

^15^AP-HP, Groupe Hospitalier “Mondor”, Pôle de Psychiatrie, 51 Avenue de Maréchal de Lattre de Tassigny, 94010 Créteil, France.

^16^INSERM, U955, Equipe 15, 51 Avenue de Maréchal de Lattre de Tassigny, 94010 Créteil, France.

^17^Faculté de Médecine, Université Paris-Est, 51 Avenue de Maréchal de Lattre de Tassigny, 94010 Créteil, France.

^18^Fondation Fondamental, 40 Rue de Mesly, 94000 Créteil, France.

^19^CMP B CHU, BP 69, 63003 Clermont Ferrand, Cedex 1, France.

^20^Université Clermont Auvergne, EA 7280, Clermont-Ferrand 63000, France.

^21^Etablissement Public de Santé Maison Blanche, Paris, France.

^22^Department of Experimental Biomedicine and Clinical Neuroscience, Section of Psychiatry, University of Palermo, Via G. La Loggia n.1, 90129 Palermo, Italy.

^23^Unit of Psychiatry, “P. Giaccone” General Hospital, Via G. La Loggia n.1, 90129 Palermo, Italy. ^24^Departamento de Neurociências e Ciencias do Comportamento, Faculdade de Medicina de Ribeirão Preto, Universidade de São Paulo, Av. Bandeirantes, 3900 -Monte Alegre- CEP 14049-900, Ribeirão Preto, SP, Brasil.

^25^Núcleo de Pesquina em Saúde Mental Populacional, Universidade de São Paulo, Avenida

Doutor Arnaldo 455, CEP 01246-903, Ribeirão Preto, SP, Brasil.

^26^Section of Psychiatry, Department of Neuroscience, Biomedicine and Movement, University of Verona, Piazzale L. A. Scuro 10, 37134 Verona, Italy.

^27^Department of Medical and Surgical Science, Psychiatry Unit, Alma Mater Studiorum Università di Bologna, Viale Pepoli 5, 40126 Bologna, Italy.

^28^Division of Psychological Medicine and Clinical Neurosciences, MRC Centre for Neuropsychiatric Genetics and Genomics, Cardiff University, Cardiff, UK.

^29^Family Genomics Research Group, Department of Biology, Maynooth University, Maynooth, Ireland.
