# Supplementary material for: The association between reasons for first using cannabis, later pattern of use, and risk of first-episode psychosis: the EU-GEI case–control study
Source: Psychol Med. 2023 May 2;53(15):7418–27. doi: 10.1017/S0033291723001071 (PMC10719678; doi:10.1017/S0033291723001071)
Supplement: Spinazzola et al. supplementary material [file S0033291723001071sup001.docx]

**SUPPLEMENTARY MATERIALS TO ACCOMPANY:**

Predictors of the reasons for first using cannabis, later pattern of use and risk of first-episode psychosis: the EU-GEI case-control study

- **1. Methods**
  - 1.1 Measures (cannabis use variables)
  - 1.2 Previous studies on motivations for cannabis use
  - 1.3 The Reasons for First Using Cannabis (RFUC) variables – the variable “other”
  - 1.4 The RFUC variables – validity and recall bias
- **2. Results**
  - 2.1 Recruitment flow chart for controls
  - 2.2 Recruitment flow chart for first-episode psychosis patients (FEPp)
  - 2.3 Descriptive sociodemographic analyses (in the whole EU-GEI case-control sample)
  - 2.4 The effect of predictors of RFUC on case-control status
  - 2.5 Missing data on the RFUC variables
  - 2.6 Different RFUC in FEP patients and in controls in our working sample
  - 2.7 The association between the time passed from age of first cannabis use until psychosis onset and the RFUC
  - 2.8 Exploring the differences in reported RFUC across the different EUGEI sites

*The supplementary material has been provided by the authors to give readers additional information about their work*

**SUPPLEMENTARY**

1. **Methods**
   1. **Measures (cannabis use variables)**

A detailed history of cannabis use and other recreational drugs was collected using an updated version of the Cannabis Experiences Questionnaire, the EU-GEI study (CEQ_EU-GEI_). Specifically, based on our previous work, we created the variables for the pattern of use, frequency of use, potency, and age at 1^st^ use, (Di Forti *et al.*, 2019).

Regarding the frequency of use, the item “how often do/did you use cannabis” originally allowed 7 possible answers: a) Only once or twice; b) About once a year; c) Few times a year; d) About once/twice a month; e) About once a week; f) More than once a week; g) Every day. These answers were grouped using three categories: a) used never or occasionally (less than once a week); b) used more than once a week (but less than daily); c) used daily. This approach was based on our previous paper, showing that only the categories “more than once a week” (OR 2.2; 95% CI 1.6 – 2.9) and “every day” (OR 6.2; 95% CI 4.8 – 8.0) had adjusted ORs greater than 1 for developing Psychotic Disorders (Di Forti *et al.*, 2019).

The cannabis potency variable was created using a cut-off of THC=10%. Participants were asked to name in their own language the name of the type of cannabis they mostly used during their period of use and the variable was then created based on the expected concentration in different types of cannabis across the study sites, as reported in the EMCDDA and by the National data on cannabis potency quoted (EMCDDA, 2017).

The low-potency cannabis category (THC<10%) included hash/resin from UK and Italy, imported herbal cannabis from the UK, Italy, Spain and France, Brazilian marijuana and hash and the Dutch *Geimporteerde Wiet*. The high-potency category (THC≥10%) included all the other types reported by the study participants in their original language street names such as UK home-grown skunk/*sensimilla* UK Super Skunk, Italian home-grown skunk/*sensimilla*, Italian Super Skunk, the Dutch *Nederwiet*, *Nederhasj* and *geimporteerde hasj*, the Spanish and French Hashish (from Morocco), Spanish home-grown *sensimilla*, French home-grown skunk/*sensimilla*/super-skunk and Brazilian skunk.

As we already mentioned in the paper, we also merged these two variables into one frequency-potency variable, as follows: occasional use with any potency of use=1, more than once a week and low potency=2, more than once a week and high potency=3, daily or almost and low potency=4, daily or almost and high potency=5. Conversely, age at first cannabis use was considered as a continuous numerical variable.

- 1. **Previous studies on motivations for cannabis use**

To confirm what we stated in the discussion, ‘*To the best of our knowledge (see supplementary materials for details of literature search), this is the first study to examine what reasons underlie first using cannabis and if these reasons are associated with later pattern of cannabis use and the risk to develop a psychotic disorder*’, we ran a search on 3 electronic libraries, MEDLINE (1950–2009), PsycINFO (1806–2009), and EMBASE (1980–2009), using the following search terms: ‘psychosis’ and ‘reasons for cannabis’.

Despite using broad search criteria, only 20 studies resulted. Of these, we excluded 6 conference abstracts and one book. Regarding the other results, none of these studies specifically assessed reasons for first using cannabis; some reported reasons/motivations for cannabis use in people with psychosis and a couple of studies compared reasons for cannabis use in patients with psychosis and in healthy controls.

The most recent study on self-reported reasons of cannabis use was published in 2022 by Leung et al. This extensive online survey was aimed at assessing the prevalence of different motivations for using medical cannabis in USA and Canada, distinguishing between physical health reasons and mental health reasons. This study did not have a case-control design and did not provide any information regarding the presence/absence of mental health diagnoses among participants (Leung *et al.*, 2022).

- 1. **The Reasons for First Using Cannabis (RFUC) variables – the variable “other”**

The RFUC items allowed subjects to provide up to 4 reasons to first start using cannabis, thus we coded each of the categories for the variable “Why did you first try cannabis?” as binary (0=NO; 1=YES). During our main analyses, we decided to exclude the variable “other” as it was difficult to interpret.

It should be mentioned that we tried to further explore how the variable “other” reasons was distributed in our sample. Indeed, it also included a free text string variable where participants were asked to illustrate why they replied, “because of other reasons”. However, those subjects who further explained the meaning of their answers were a minority. In **Figure 1** we reported a flow chart indicating the distribution of the RFUC “other”.

**Figure 1.** *Indicating how the variable RFUC because of “other reasons” was distributed in our sample. It also included a free text string variable where participants were asked to illustrate why they replied because of “other reasons”. Those responses were subdivided into different categories.*

**
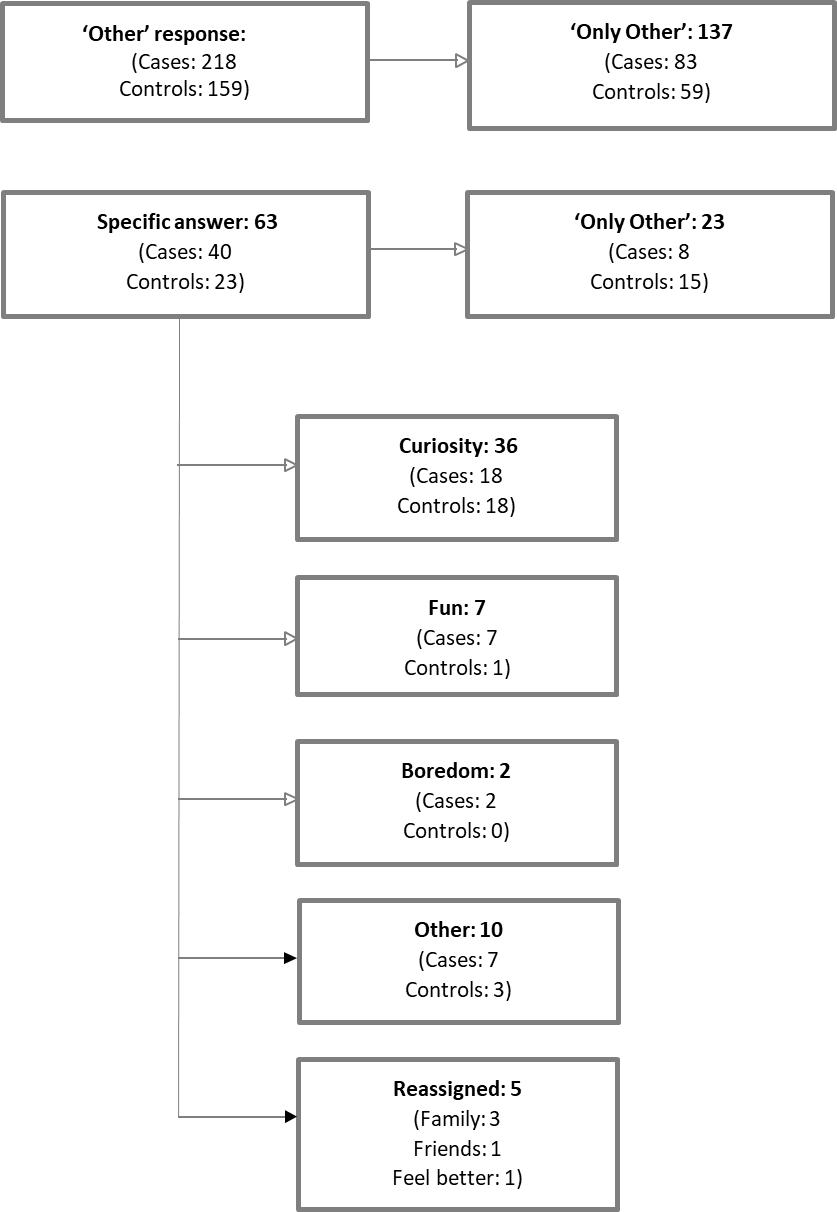
**

**1.3 The RFUC variables – validity and recall bias**

As we mentioned in the paper, the information on RFUC was derived from the responses to the question “why did you first try cannabis?” in the form of the following multiple-choice: a) my friends were using it; b) my family members were using it; c) to feel better (to get relief from either physical or psychological discomfort); and d) other. This question is part of the Cannabis Experiences Questionnaire (CEQ) which is a validated questionnaire used in many other peer-reviewed publications. However, since it is the first study to address specifically this variable, we conducted further analyses comparing the baseline and the follow-up data both from the Genetic and Psychosis (GAP) study and from the London sample of the EU-GEI study, to rule out possible recall bias issues. We found good level of agreement for all our variables both in cases and controls. See **Table 1**.

**Table 1.** *Testing the reliability for our three different RFUC variables*

|  | | Kappa coefficient |
| --- | --- | --- |
| RFUC “friends” | Controls | Κ = 0.9 |
|  | Cases | Κ = 0.79 |
| RFUC “family” | Controls | Κ = 0.89 |
|  | Cases | Κ = 0.91 |
| RFUC “to feel better” | Controls | Κ = 0.87 |
|  | Cases | Κ = 0.72 |

1. **Results**
   1. **Recruitment flow chart for controls**

**Figure 2.** *Recruitment flow chart for controls*


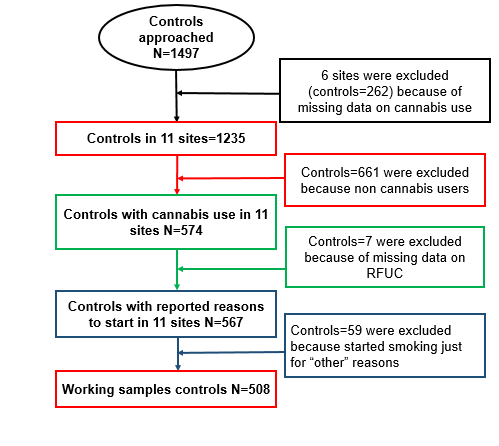


**2.2 Recruitment flow chart for FEPp**

**Figure 3.** *Recruitment flow chart for cases*


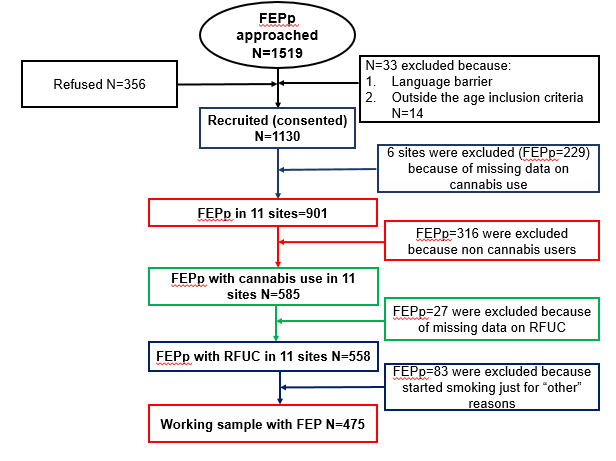


- 1. **Descriptive sociodemographic analyses (in the whole EU-GEI case-control sample)**

**Table 2.** *Descriptive sociodemographic analyses (in the EU-GEI case-control sample)*

|  | | **Controls Mean (SD)/ n (%)** | **Cases Mean (SD)/n (%)** | **Statistics** | **p value** |
| --- | --- | --- | --- | --- | --- |
| **FEPp – Control** | | 1235 (57.82) | 901 (42.18) | | |
| **Age, years** | | 36.18 (13.40) | 30.76 (10.51) | U = 9.09 | p < 0.001 |
| **Gender** | **Male** | 580 (47.00) | 558 (61.93) | χ^2^ = 46.63 | p < 0.001 |
| **Ethnicity** | **White** | 927 (75.18) | 532 (59.11) | χ^2^ = 62.17 | p < 0.001 |
|  | **All others** | 306 (24.82) | 368 (40.89) |  |  |
| **Cannabis use** | | 574 (46.97) | 585 (65.88) | χ^2^ = 74.25 | p < 0.001 |
| **Years of education** | | 14.84 (4.15) | 13.09 (4.12) | U = 9.59 | p < 0.001 |
| **Unemployment** | | 512 (41.83) | 413 (53.15) | χ^2^= 24.51 | p < 0.001 |
| **Marital status (single)** | | 458 (37.15) | 550 (64.63) | χ^2^ = 152.30 | p < 0.001 |
| **No independent living** | | 420 (34.20) | 452 (57.65) | χ^2^ = 107.16 | p < 0.001 |
| **Tobacco use** | **>11 per day** | 134 (11.07) | 262 (30.22) | χ^2^ = 120.17 | p < 0.001 |
| **Parental mental illness** | **Yes** | 304 (24.90) | 330 (37.54) | χ^2^ = 38.77 | p < 0.001 |
| **Parental psychosis** | **Yes** | 48 (3.93) | 103 (11.72) | χ^2^ = 46.43 | p < 0.001 |

*t = t-test; χ^2^ = Chi-squared test; *p-value≤0.05*

- 1. **The effect of predictors of RFUC on case-control status**

**Table 3.** *The effect of predictors of RFUC on case-control status (in cannabis users with reported RFUC)*

|  | | **Total, Mean (SD) / N (%)** | **Controls, Mean (SD) / N (%)** | **Cases, Mean (SD) / N (%)** | **Controls vs Cases Statistics** | **p value** |
| --- | --- | --- | --- | --- | --- | --- |
| **Ethnicity** | White | 802 (71.42) | 464 (81.98) | 338 (60.68) | χ^2^ = 62.37 | p < **0.001** |
|  | All others | 321 (28.58) | 102 (18.02) | 219 (39.32) |  |  |
| **IQ** | | 96.71 (20.20) | 105.33 (17.55) | 86.66 (18.37) | U = 14.97 | p < **0.001** |

*χ^2^ = Chi-squared test; U = Mann-Whitney U test*

- 1. **Missing data on the RFUC variables**

As reported in the main text, we had missing data on RFUC for 27 FEPp (4.62%) and 7 controls (1.22%). We compared the sample with complete data on RFUC with the one including the FEPp cases and controls with the missing data. As reported in **Table 2,** the two samples did not differ for age, gender and ethnicity. Therefore, we carried out the analyses using the dataset with complete data on RFUC rather than manipulating data through multiple imputation approaches. In addition, we excluded from our analyses 59 controls and 83 cases, thus resulting in 10.41% of controls and 14.87% of FEPp that were excluded from our final analyses. We carried further analyses to compare the working sample (RFUC “friends”, “family”, or “to feel better”) with the excluded subjects (only “other” RFUC). See **table 3**.

**Table 4.** *Descriptive sociodemographic analyses for comparisons between participants with data on RFUC (N=1125; 567 controls and 558 FEPp) and those with missing data on reasons to start (N=34; 7 controls and 27 FEPp), in subjects with reported cannabis use*

|  | | **Complete Dataset on RFUC**  **Mean / n (% or SD)** | **Missing data on RFUC Mean /n (% or SD)** | **Statistics** | **p value** |
| --- | --- | --- | --- | --- | --- |
| **Age, years** | | 31.20 | 31.06 | U= - 0.29 | p=0.77 |
| **Gender** | Male | 700 (62.22) | 23 (67.65) | χ^2^(1) =0.41 | p=0.52 |
| **Ethnic minority status** | Yes | 321 (28.58) | 12 (35.29) | χ^2^(2) = 0.72 | p=0.39 |
| **IQ score** | | 96.70 (20.20) | 93.33 (19.45) | U = 0.50 | p=0.62 |

*U = t-test; χ^2^ = Chi-squared test*

**Table 5.** *Descriptive sociodemographic analyses for comparisons between the excluded subjects who replied only “other” RFUC (N=142; 59 controls and 83 FEPp), and the working sample of those who reported either “friends”, “family”, or “to feel better” as their RFUC (N=983; 508 controls and 475 FEPp)*

|  | | **Working Dataset**  **Mean / n (% or SD)** | **“Other” RFUC Mean /n (% or SD)** | **Statistics** | **p value** |
| --- | --- | --- | --- | --- | --- |
| **Age, years** | | 31.51 (10.89) | 29.07 (10.73) | U = 3.18 | p=**0.002** |
| **Gender** | Male | 604 (61.44) | 96 (67.61) | χ^2^(1) = 2 | p=0.16 |
| **Ethnic minority status** | Yes | 280 (28.51) | 41 (29.08) | χ^2^(2) = 0.019 | p=0.89 |
| **IQ score** | | 96.85 (20.19) | 95.79 (20.31) | U = 0.63 | p=0.53 |

*U = Mann-Whitney U test*

**2.6 Different RFUC in FEP patients and in controls in our working sample**

**Figure 4.** *Number of cases, controls, and total number reporting the different RFUC*

**2.7 The association between the time passed from age of first cannabis use until psychosis onset and the RFUC**

As we mentioned in the discussion, we also conducted further analyses to see whether, in FEPp, the time gap between age at first cannabis use and age of psychosis onset was associated with different RFUC. We were particularly interested in the association between this time gap and the RFUC “to feel better”, as it could indirectly suggest a sign of self-medication. First, we conducted a series of Mann-Whitney tests (t-test where appropriate) to see whether there was an association between age at first cannabis use and RFUC and between age at first psychosis presentation and RFUC (Table 3). Then we generated two different variables 1) time gap between age at first cannabis use and age of psychosis onset, and 2) the time gap between age at first cannabis use and age at first psychosis presentation. We compared these variables with the different RFUC through simple comparisons (Table 3) and linear regressions (Table 4). As can be seen in the tables, those who reported RFUC “to feel better” appear to have a slightly, non-significant, lower gap time between age at first cannabis use and age at first psychosis presentation; however, all groups have an average of psychosis onset at least ten years later than the first use of cannabis. In addition, these analyses can also help to validate our path model, thus confirming the temporal relationship between our main variables.

**Table 6.** *Analyses conducted just in the subgroups of subjects with FEP showing the associations between the time passed from age of first use of cannabis until psychosis onset and the RFUC*

|  | **“Friends” yes/no N (SD)** | **Statistics and p value** | **“Family” yes/no N (SD)** | **Statistics and p value** | **“To feel better” yes/no N (%)** | **Statistics and p value** |
| --- | --- | --- | --- | --- | --- | --- |
| **Age at first cannabis use** | 420 (6.07) | U = 0.54  p = 0.59 | 68 (6.75) | U = 4.18  p <0.001 | 112 (4.57) | U = -0.15  p = 0.88 |
| **Age at first presentation** | 422 (8.56) | U = -2.19  p = **0.03** | 69 (8.78) | U = 0.27  p = 0.79 | 112 (7.99) | U = 1.01  p = 0.31 |
| **Time gap between age at first cannabis use and psychosis onset** | 420 (7.97) | U = -3.19  p = **0.001** | 68 (7.73) | U = -1.25  p = 0.21 | 112 (7.02) | U = 1.24  p = 0.22 |
| **Time gap between age at first cannabis use and psychosis presentation** | 420 (7.98) | U = -3.25  p = **0.001** | 68 (7.78) | U = -1.17  p = 0.24 | 112 (7.09) | U = 1.24  p = 0.22 |

*U = Mann-Whitney U test*

**Table 7.** *Linear regression analyses conducted just in the subgroups of people with FEP* *showing the associations between the time passed from age of first use of cannabis until psychosis onset and the RFUC*

|  | **Friends**  β (95% CI) | **Family**  β (95% CI) | **Better**  β (95% CI) |
| --- | --- | --- | --- |
| **Time gap between age at first cannabis use and psychosis onset** | **2.27 (0.77 – 3.78)** | 0.86 (-1.13 – 2.85) | -1.33 (-2.95 – 0.29) |
| **Time gap between age at first cannabis use and psychosis presentation** | **2.29 (0.78 – 3.81)** | 0.79 (-1.21 – 2.80) | -1.32 (-2.95 – 0.31) |

**2.8 Exploring the differences in reported RFUC across the different EUGEI sites**

We tried to explore differences in reported RFUC in different sites across the EUGEI, as this might suggest a normalisation of cannabis use or tolerance of use in some sites/countries. We did find significant differences (χ^2^=32.67; p<0.001). Interestingly, in Amsterdam and in Gouda/Voorhout, the only two sites in the EUGEI where cannabis is legal (The Netherlands), the number of participants who reported having started using cannabis because of family members was not high (Amsterdam = 6.67%, Gouda/Voorhout = 10.57%). It was certainly lower than in Paris (15.94%), Southeast London (14.39%), and Ribeirao Preto (13.07%). This finding seems to indicate that the legalisation of cannabis use does not increase the chances of starting to use cannabis in the family environment. Apart from this, there do not seem to be any noticeable differences in RFUC and our different sites (see Tables 8, 9 and 10).

**Table 8.** *Exploring the differences in reported RFUC “because of friends” across the different EUGEI sites*

| **Site** | **“Friends”**  YES N (%) | **Statistics** |
| --- | --- | --- |
| London | 228 (84.1) | χ^2^(10) = 48.0; p < 0.001 |
| Cambridgeshire | 65 (81.3) |  |
| Amsterdam | 95 (63.3) |  |
| Gouda and Voorhout | 92 (74.8) |  |
| Madrid | 36 (80.0) |  |
| Barcelona | 41 (93.2) |  |
| Paris | 56 (81.2) |  |
| Puy de Dome | 30 (96.8) |  |
| Bologne | 67 (90.5) |  |
| Palermo | 78 (83.8) |  |
| Ribeirao Preto | 122 (80.3) |  |
| Total | 910 (80.4) |  |

**Table 9.** *Exploring the differences in reported RFUC “because of family members” across the different EUGEI sites*

| **Site** | **“Family”**  YES N (%) | **Statistics** |
| --- | --- | --- |
| London | 39 (14.4) | χ^2^(10) = 32.7; p < 0.001 |
| Cambridgeshire | 7 (8.8) |  |
| Amsterdam | 10 (6.7) |  |
| Gouda and Voorhout | 13 (10.6) |  |
| Madrid | 2 (4.4) |  |
| Barcelona | 1 (2.27) |  |
| Paris | 11 (15.9) |  |
| Puy de Dome | 1 (3.2) |  |
| Bologne | 3 (4.1) |  |
| Palermo | 0 (0) |  |
| Ribeirao Preto | 20 (13.1) |  |
| Total | 107 (9.4) |  |

**Table 10.** *Exploring the differences in reported RFUC “to feel better” across the different EUGEI sites*

| **Site** | **“Better”**  YES N (%) | **Statistics** |
| --- | --- | --- |
| London | 42 (15.5) | χ^2^(10) = 71.5; p < 0.001 |
| Cambridgeshire | 6 (7.5) |  |
| Amsterdam | 10 (6.7) |  |
| Gouda and Voorhout | 7 (5.7) |  |
| Madrid | 8 (17.8) |  |
| Barcelona | 4 (8.9) |  |
| Paris | 10 (14.5) |  |
| Puy de Dome | 4 (12.9) |  |
| Bologne | 4 (5.4) |  |
| Palermo | 3 (3.2) |  |
| Ribeirao Preto | 47 (30.7) |  |
| Total | 145 (12.8) |  |

**Supplementary methods reference:**

**Di Forti, M., Quattrone, D., Freeman, T. P., Tripoli, G., Gayer-Anderson, C., Quigley, H., Rodriguez, V., Jongsma, H. E., Ferraro, L. & La Cascia, C.** (2019). The contribution of cannabis use to variation in the incidence of psychotic disorder across Europe (EU-GEI): a multicentre case-control study. *The Lancet Psychiatry* **6**, 427-436.

**EMCDDA, I.** (2017). European drug report 2017: trends and developments. *Luxembourg: Publications Office of the European Union*.

**Leung, J., Chan, G., Stjepanović, D., Chung, J. Y. C., Hall, W. & Hammond, D.** (2022). Prevalence and self-reported reasons of cannabis use for medical purposes in USA and Canada. *Psychopharmacology* **239**, 1509-1519.
